# Molecular Mechanisms of HIV Immune Evasion of the Innate Immune Response in Myeloid Cells

**DOI:** 10.3390/v5010001

**Published:** 2012-12-21

**Authors:** Mike Mashiba, Kathleen L. Collins

**Affiliations:** 1 Program in Immunology, University of Michigan, Ann Arbor, MI 48109, USA; E-Mail: mmashiba@umich.edu; 2 Medical Scientist Training Program, University of Michigan, Ann Arbor, MI 48109, USA; 3 Department of Internal Medicine, University of Michigan, Ann Arbor, MI 48109, USA; 4 Department of Microbiology and Immunology, University of Michigan, Ann Arbor, MI 48109, USA; 5 Program in Cellular and Molecular Biology, University of Michigan, Ann Arbor, MI 48109, USA

**Keywords:** HIV, immune evasion, Vpr, Ung2, myeloid

## Abstract

The expression of intrinsic antiviral factors by myeloid cells is a recently recognized mechanism of restricting lentiviral replication. Viruses that enter these cells must develop strategies to evade cellular antiviral factors to establish a productive infection. By studying the cellular targets of virally encoded proteins that are necessary to infect myeloid cells, a better understanding of cellular intrinsic antiviral strategies has now been achieved. Recent findings have provided insight into how the lentiviral accessory proteins, Vpx, Vpr and Vif counteract antiviral factors found in myeloid cells including SAMHD1, APOBEC3G, APOBEC3A, UNG2 and uracil. Here we review our current understanding of the molecular basis of how cellular antiviral factors function and the viral countermeasures that antagonize them to promote viral transmission and spread.

## 1. Introduction

### Intrinsic Anti-Viral Factors Limit Infection of Myeloid Cells by HIV and SIV

Myeloid cells, including dendritic cells and macrophages, play an important role in the innate and adaptive immune response against viral pathogens like HIV. Myeloid cells are also important targets of HIV and SIV [1,2]. Macrophages and dendritic cells (DCs) express the necessary receptors (CD4 and chemokine co-receptor(s)) required for HIV-1 entry and, like CD4^+^ T cells [3,4], are amongst the earliest targets for HIV-1 and SIV *in vivo* [5,6], (reviewed in [7]). HIV and SIV have been detected in macrophages in secondary lymphoid tissue by *in situ* hybridization *in vivo* [8,9]. Moreover, at later stages of pathogenesis, HIV infected macrophages are thought to be the cause of AIDS related encephalopathy [10], and SIV infected macrophages cause an analogous central nervous system pathology in the rhesus macaque model [11]. However, myeloid cells are somewhat resistant to HIV and SIV infection because they express high levels of host restriction factors that represent significant post-entry blocks to HIV-1 infection [12,13].

DCs propagate HIV-1 primarily by *trans* infection, a pathway in which DCs capture and transmit internalized viral particles by C-type lectin receptors, a family that includes DC-specific intercellular adhesion molecule 3-grabbing nonintegrin (DC-SIGN), and mannose binding C-type lectin receptors (MCLR) [14,15]. A heparin sulfate proteoglycan dependent pathway and a cholesterol-dependent pathway for internalization of intact viral particles have also been described [16,17]. More recently, it was shown that sialyllactose is a molecular recognition pattern in gangliosides in the HIV-1 membrane that allows DCs to capture viral particles [18,19]. In addition, galactosyl ceramide can mediate cell to cell transfer of HIV-1 from dendritic cells to T lymphocytes [20]. 

Productive infection of DCs with HIV-1 has also been reported *in vitro* [21]. However, only a small percentage of DCs have been found to be infected *in vivo* [22] and most evidence indicates that DCs do not replicate HIV efficiently. Therefore, the contribution of HIV infection of DCs to pathogenesis requires further study. It is possible that the main role of DCs in HIV disease is to transmit internalized viral particles to CD4^+^ T cells rather than to directly support productive infection [14,23] (reviewed in [24,25]). 

Within the myeloid lineage, macrophages are thought to be the most permissive to HIV-1. Macrophages become permissive to HIV-1 following differentiation because of a decrease in the expression of host restriction factors [12,13]. In addition to being infected, there is also evidence that macrophages archive HIV-1 virions for transfer to CD4^+^ T lymphocytes via the virological synapse, an HIV-induced interface between two cells that facilitates cell to cell infection [26]. Infection of macrophages may be particularly important because, compared to HIV-infected T cells, infected macrophages have a relatively long half-life [26]. In sum, current data indicates that myeloid cells play an important role in the pathogenesis of HIV-1 infection as a relatively long-lived target of HIV and as a viral conduit to CD4^+^ T cells.

## 2. Viral Factors Counteract Intrinsic Antiviral Factors; the Role of Vpx in SIV Pathogenesis

**Figure 1:**
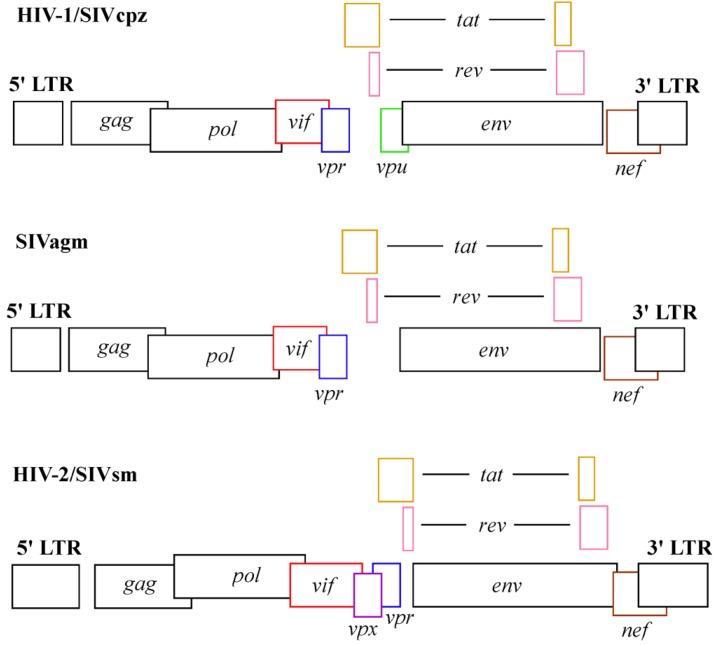
Genomic organization of HIV-1/SIVcpz, SIVagm and HIV-2/SIVsm [32]. HIV‑1 contains the *vpu* accessory gene, but not the vpx accessory gene. HIV-2 and SIVsm encode *vpx* but not *vpu*. SIVagm encodes neither *vpu* nor *vpx*.

HIV-1, HIV-2 and SIV contain accessory proteins that promote infection of myeloid cells: HIV-2 and simian immunodeficiency virus of sooty mangabeys (SIVsm) encode Vpr and Vpx, a protein that originated from Vpr [27], while HIV-1 only encodes Vpr [28] (reviewed in [29,30,31,32], Figure 1). SIVs related to HIV-2/SIVsm lacking Vpx (Δv*px*) are significantly less efficient than wildtype SIVs at establishing an infection in the pigtail macaque model by intrarectal or intravenous inoculation [33,34]. Animals infected with SIV Δ*vpx* maintain lower viral loads and higher CD4^+^ T cell counts than animals infected with wildtype SIV. However, Rhesus monkeys infected with SIV Δ*vpx* eventually acquire AIDS related symptoms, indicating that while Vpx promotes infection, it may not be absolutely required for pathogenesis *in vivo* [35]. 

### 2.1. *In vivo* Studies of Vpx Function

A number of studies have been performed to better define the activities of Vpx *in vivo*. One such study examined SIVmne027 infection in pigtail macaques plus or minus the expression of a functional Vpx protein [33]. Seven to 10 days following intrarectal inoculation, wildtype SIVmne027 infected macaques had viral loads almost 100 fold higher than SIVmne027 Δ*vpx* infected animals. CD4^+^ T cell counts were reduced from baseline in wildtype SIVmne027 infected animals, but not in SIVmne Δ*vpx* infected animals. Based on *in situ* hybridization, 98% of infected cells found in secondary lymphoid tissue were lymphocytes, whereas infected myeloid cells were rare (fewer than 2% of infected cells were HAM56+ macrophages and no infected DCs were found) [33]. The dramatic effect of Vpx on infection of CD4^+^ T cells *in vivo* seems to contrast with the *in vitro* results that indicate a primary role for Vpx in the infection of myeloid cells (see below) [33]. Further studies are clearly needed to better understand this apparent enigma. 

### 2.2. *In vitro* Studies of Vpx Function

In cell culture models, Vpx has only a minimal effect on infection of primary T cells whereas Vpx dramatically enhances lentiviral infection of macrophages. The effect of Vpx on infectivity correlates with enhanced accumulation of 2-LTR circles (dead-end HIV-1 cDNAs measured because they are proportional to the amount of nuclear HIV-1 cDNA [36,37]). This activity of Vpx requires expression of the host protein, damaged DNA binding protein 1 (DDB1), the E3 ubiquitin ligase complex scaffolding factor previously shown to be required for Vpr to cause cell cycle arrest [37]. It was proposed that the interaction of Vpx with DDB1 led to the degradation of host proteins that are detrimental to viral replication [2,37]. 

#### 2.2.1. Vpx Targets the Host Factor SAMHD1

Recent studies have now demonstrated that Vpx promotes macrophage and DC infection by targeting the cellular factor, SAMHD1 [38,39] (also reviewed in [40,41]). SAMHD1 was initially identified as a host protein that bound to Vpx [38,39]. Subsequent studies demonstrated that silencing SAMHD1 enhanced HIV-1 and SIV Δ*vpx* infection of myeloid cells [38,39]. SAMHD1 silencing also enhances HIV-1 infection of resting CD4^+^ T cells, suggesting that SAMHD1 also restricts HIV-1 infection in quiescent T cells [42,43]. Conversely, ectopic overexpression of SAMHD1 reduced HIV-1 and SIV Δ*vpx* infection of otherwise permissive cell lines [39]. Based on these data, SAMHD1 is necessary and sufficient to inhibit infection of myeloid cells and quiescent CD4^+^ T cells by lentiviruses not expressing Vpx [38,39]. 

**Figure 2:**
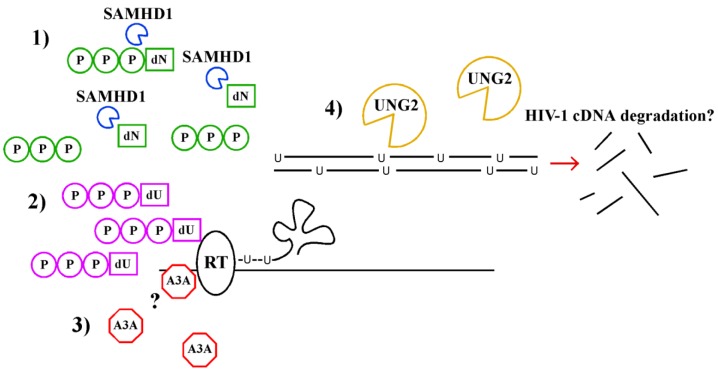
Mechanisms in myeloid cells that inhibit HIV-1 reverse transcription. dNTPs are dephosphorylated by SAMHD1 (**1**). A high intracellular concentration of dUTP relative to dTTP in myeloid cells increases the incorporation of deoxyuridine into HIV-1 cDNA by reverse transcriptase (**2**). A3A deaminates cytidine to uridine in HIV-1 cDNA (**3**). A question mark (?) indicates that A3A may slow reverse transcription through an unknown mechanism. UNG2 catalyzes the removal of uracil from HIV-1 cDNA, and may lead to fragmentation or degradation of HIV-1 cDNA (**4**).

SAMHD1 contains a putative HD domain that provided investigators with some clues as to the role of SAMHD1 in lentiviral infection [39]. HD domains contain conserved histidine and aspartate catalytic residues and are found within a superfamily of metalloenzymes with known or predicted phosphohydrolase activity [44]. Studies by Goldstone *et al.* [45] and Lahouassa *et al.* [46] have recently provided evidence that SAMHD1 inhibits HIV-1 infection in myeloid cells by restricting the intracellular pool of dNTPs (highlighted in [47] and [48], respectively, Figure 2). Recombinant SAMHD1 reduces the concentration of all four dNTPs by direct hydrolysis *in vitro* [45,46] and expression of, SAMHD1 in myeloid cells reduces dNTP concentrations to a level that is suboptimal for reverse transcription [46]. Silencing SAMHD1, providing Vpx in trans and provision of exogenous deoxynucleosides all increase the amount of available dNTP [46] and dramatically increase myeloid cell permissivity to HIV-1 and SIVΔ*vpx*. Thus, the main mechanism employed by SAMHD1 to restrict lentiviral infection is dNTP hydrolysis. 

Given the striking ability of SIV Vpx to stimulate HIV-1 infection of myeloid cells by inhibiting SAMHD1, it is puzzling as to why HIV-1 appears to lack an equivalent activity against SAMHD1. Some have suggested that myeloid cells play a limited role in the propagation of HIV-1 *in vivo* because CD4^+^ T cells are more permissive to HIV [48]. Others predict that infection of some types of myeloid cells by HIV would be detrimental to the overall infection [48]. This hypothesis is supported by experiments in which coerced infection of dendritic cells treated with Vpx containing VLPs was found to trigger an IFN response that inhibited the infection of other cell populations [49]. Despite this hypothesis, HIV-1 infected CD11c^+^ macrophages are frequently found in lymph node biopsies from chronically infected individuals [50], and any anti-viral cytokines secreted by these cells are insufficient to contain the infection. Thus, additional experiments will be needed to better understand whether SAMHD1 has an effect on the infection of myeloid cells during HIV-1 infection *in vivo*. 

#### 2.2.2. Vpx Counteracts SAMHD1 via the Ubiquitin Ligase Complex DDB1 and CUL4A

Recent studies have demonstrated that Vpx counteracts SAMHD1 by targeting the restriction factor for degradation [38,39]. In Vpx expressing cells, SAMHD1 interacts with the ubiquitin ligase complex DDB1-CUL4A-DCAF E3 [38,39]. Vpx-dependent degradation of SAMHD1 is prevented by silencing DCAF1 or by inhibiting proteasomal degradation [38,39]. These studies support the model that Vpx degrades SAMHD1 by promoting its interaction with the DDB1-CUL4-DCAF E3 ubiquitin ligase complex, which promotes SAMHD1 ubiquitylation and proteasomal degradation. 

Interestingly, Vpx stimulates infectivity in primary human macrophages to a greater extent than SAMHD1 knockdown [46]. While this observation might be explained by partial knockdown of SAMHD1, it is also possible that SAMHD1 is not the only target of Vpx that is relevant in myeloid cells. Indeed, it has been reported that Vpx causes partial degradation APOBEC3A, another potential host restriction factor in macrophages [12].

## 3. Viral Factors Counteract Intrinsic Antiviral Factors; the Role of Vpr in HIV and SIV Infection

In comparison to Vpx, which is encoded by only two lentiviral lineages, Vpr is conserved amongst all primate lentiviruses [27]. However, a requirement for Vpr is harder to demonstrate *in vitro* and is more prominent at low multiplicity of infection (MOI) [51]. While the major effect of Vpr seems to be to enhance infection of macrophages there is also evidence Vpr can enhance the infection of activated CD4^+^ T lymphocytes under some conditions [21,52,53,54]. 

### 3.1. *In vivo* Studies of Vpr Function in SIV Infected Macaques

To examine the role of Vpr, investigators have utilized rhesus macaques infected with SIVmac239, which causes AIDS like symptoms. In these studies, an SIV containing a mutation in the Vpr start codon (SIVmac239 *vpr^−^*) reverted to wild type in 3 of 5 animals [55]. Low viral load was observed in the two animals infected with virus that did not revert. However, in a subsequent study, using an SIVmac239 *vpr^−^* deletion mutant that cannot revert *in vivo*, 2 of 4 animals infected with SIV Δ*vpr* developed AIDS like symptoms [56]. Therefore, Vpr may not be absolutely required for the development of AIDS in this model system.

### 3.2. The Role of Vpr in HIV Pathogenesis

Unlike SIV, HIV does not contain Vpx and does not require Vpx for infection *in vivo*. Vpr is contained within HIV and is more conserved among primate lentiviruses than Vpx [27]. However, Vpr has a comparably modest effect on macrophage and dendritic cell infection [21,52,54]. While *in vivo* studies have not been able to directly address the contribution of Vpr to HIV-1 pathogenesis, a Vpr allele with attenuated cytopathogenicity *in vitro* has been isolated from a long-term non-progressor [57]. These findings suggest that Vpr is important for HIV replication *in vivo*.

### 3.3. *In vitro* Studies of Vpr Function

Further investigation is needed to understand how Vpr stimulates infection. Some SIV Vpr alleles are able to degrade SAMHD1 in a species-specific manner. However all HIV-1 Vpr alleles that have been tested lack activity against human SAMHD1 [27]. The interaction of Vpr and DDB1 results in the ubiquitin mediated proteasomal degradation of UNG2 [58]. However, the role of UNG2 has been controversial with studies showing both positive and negative effects, as discussed below. Thus, Vpr may also target an additional unknown cellular factor to mediate its full complement of functions.

#### Uracil as a Restriction Factor

Non-dividing cells, such as macrophages, have been reported to have a high dUTP/dTTP ratio relative to CD4^+^ T lymphocytes [59]. In macrophages, the intracellular ratio of dUTP to dTTP is sufficient to allow uracil incorporation into viral cDNA, while the dUTP to dTTP ratio in PBMCs is not [59] (Figure 2). If uracil incorporation into viral DNA restricts viral replication (reviewed in [60]), the dUTP/dTTP ration in macrophages predicts this restriction would be more efficient in macrophages than in CD4^+^ T lymphocytes. 

In a separate study, that measured incorporation of uracil into HIV DNA (uracilation) in primary T cells 48 hours post infection, uracil was only detected in the absence of both Vpr and Vif expression [61] (highlighted in [62]) and was not apparent in wild type virus. To detect uracil incorporation, this study utilized uracil DNA glycosylase (UDG), which removes the uracil base from uridine containing DNA rendering uracilated templates inactive for subsequent PCR amplification. Because uracilation was only detectable in the absence of Vif and Vpr, it was hypothesized that both Vif and Vpr play a role in reducing uracil incorporation. Vif acts by inhibiting the host restriction factor, APOBEC3G (A3G), a cytosine deaminase, which increases uracil incorporation in single stranded viral DNA during reverse transcription by deaminating cytosine residues [63]. Vpr may reduce uracil incorporation by activating DNA repair pathways [61]. As described above, Vpr is known to interact with UNG2, a cellular uracil glycosylase, which normally functions to remove uracil incorporated into DNA [58,64]. Vpr also activates ATM and ATR DNA damage signaling pathways [65], which in turn stimulate repair of DNA (reviewed in [66,67]). Interestingly, macrophages do not appear to express ATR [65]. If ATR is necessary for the repair of uracil containing DNA, then uracilation of the HIV genome may be a more important restriction factor in macrophages than in T cells. 

Data from other studies have provided evidence that UNG2, is recruited into the viral particle and is necessary for HIV cDNA stability [68]. The authors of these studies used the differential ability of Taq and Pfu polymerases to demonstrate the presence of uracil containing templates. These studies revealed that depletion of UNG2 in virus-producing cells substantially increases uracil incorporation into early reverse transcripts in the target cell. In addition, these authors used a synthetic uracil‑containing primer-template substrate to demonstrate that recombinant UNG2 and HIV-1 RT can cooperate to repair uracilated DNA in a cell-free system. These data suggest that UNG2 supports viral replication by catalyzing repair of uracilated viral cDNA.

There is also evidence that UNG2-dependent uracil deglycosylation is detrimental to viral infection (reviewed in [69], Figure 2). In support of this model, one study demonstrated that expression of the UNG2 inhibitor, Ugi, or silencing of UNG2 in virus producing cells partially blocks the antiviral activity of A3G against mutant HIVs that lack *vif^−^* [70]. Thus, UNG2-dependent deglycosylation of hyperuracilated DNA and subsequent cleavage by apurinic endonucleases may lead to viral cDNA degradation or inhibition of reverse transcription [70]. 

Another recent study observed positive effects of uracilation on overall HIV infection. These authors measured uracil incorporation at 10 hours post infection, before integration has occurred and provided evidence that uracilation promotes integration by limiting the nonproductive autointegration pathway that produces two LTR circles [71]. To measure uracilation, these investigators took advantage of the differential abilities of Taq and Pfu to amplify a uracilated template. In addition, an *in vitro* integration assay was employed to measure the efficiency of integration of DNA containing varying amounts of uracil. In this assay, less autointegration was detected when templates contained more uracil. Thus, this study concluded that uracil incorporation into HIV cDNA can actually have a beneficial role in HIV infection by inhibiting non-productive auto-integration pathways.

## 4. Viral Factors Counteract Intrinsic Antiviral Factors; Vpx and Vif Counteract APOBEC Family-Mediated Restriction

Vif, an HIV accessory protein, protects the viral genome by degrading the host restriction factor A3G [72]. In the absence of Vif, A3G is packaged within viral particles and renders them non‑infectious in the next round of replication through hypermutation of viral cDNA or inhibition of reverse transcription [73]. A3G is expressed in a wide range of cell types that includes the primary targets of HIV-1, CD4^+^ T lymphocytes and macrophages [74]. 

Increasing evidence suggests that A3G is a member of a family of cytidine deaminases that plays a role in host innate immunity (reviewed in [75]). It was recently discovered that APOBEC3A (A3A), one member of this family, is highly expressed in myeloid cells. The high level expression of A3A in immature monocytic cells contributes to their resistance to HIV infection [12,76] (Figure 2). Upon differentiation of monocytic cells into more mature macrophages, A3A expression decreases [76]. Remarkably, it was found that silencing A3A in monocytes infected with HIV-1 GFP reporter increases virus particle production 3 to 4 fold [76]. Similarly, it was reported that A3A silencing increases HIV infection of primary macrophages, dendritic cells, and the monocytic cell line, THP-1, by 5–7 fold [12]. In the same study, the authors demonstrate that Vpx reduces A3A expression in 293T cells co-transfected with plasmids expressing A3A and SIV_MAC_ Vpx. Based on this data it appears that A3A may be an additional target of Vpx that leads to increased permissivity of myeloid cells to lentiviruses.

There is a great deal of evidence that most A3 family members act within the viral particle such that A3 expression in the viral producer cells determines the requirement for Vif. However, recent studies have provided evidence that in some cases A3 expression in target primary macrophages may also play a role [77]. Because A3 proteins favor certain target sequences for deamination, it is possible to link patterns of mutation to particular A3 family member activity. While A3G targets 5'-CC sequences for deamination on the (−) strand, leading to 5'-AG hypermutations on the (+) strand, the other APOBEC proteins preferentially deaminate the 5'-TC dinucleotide sequence (reviewed in [78]). Because an analysis of HIV DNA amplified from macrophages using PCR revealed G to A hypermutation with a strong 5'-TC (−) strand bias, the authors concluded that a non-A3G APOBEC protein was playing a role. In sum, these studies indicate that A3 family members are capable of acting on HIV in the target cell and that A3A is a strong candidate for this role in macrophages [12]. However, because only 1% to 6% of HIV-1 sequences amplified contained G to A hypermutations, this study also concluded it is unlikely that A3A mediated hypermutation accounts for A3A mediated lentiviral restriction in myeloid cells and propose that A3A has other mechanisms to inhibit HIV (Figure 2) [77]. We may be only beginning to understand the antiviral properties of A3 proteins. 

## 5. Conclusion

Macrophages and DCs are important cell types in the pathogenesis of SIV and HIV because they are early targets for infection [5] and long-lived virus-producing cells capable of promoting viral transfer to CD4^+^ T cells [26]. Thus, it is important to understand innate immunity to lentiviral infection in these cell types. Recent evidence suggests that myeloid cells have unique innate immune factors that counteract lentiviral infection, including high expression of the myeloid-specific host restriction factors (SAMHD1 and A3A) and a high intracellular dUTP/dTTP ratio. It is still unclear whether or how these mechanisms cooperate: Does SAMHD1 nucleotide hydrolysis elevate the dUTP/dTTP ratio? What is the relative impact of uracil incorporation by A3A deamination compared with misincorporation of dUTP by reverse transcriptase? Does the increased time required for reverse transcription in the presence of SAMHD1 and low concentrations of dNTPs make the preintegration complex more susceptible to deamination by A3A? A better understanding of the relative importance of each of these pathways and their interplay is crucial for the development of new therapies to enhance innate immunity to HIV infection in myeloid cells. 

## References

[1] Cameron P.U., Forsum U., Teppler H., Granelli-Piperno A., Steinman R.M. (1992). During hiv-1 infection most blood dendritic cells are not productively infected and can induce allogeneic cd4+ t cells clonal expansion. Clin. Exp. Immunol..

[2] Kaushik R., Zhu X., Stranska R., Wu Y., Stevenson M. (2009). A cellular restriction dictates the permissivity of nondividing monocytes/macrophages to lentivirus and gammaretrovirus infection. Cell Host Microbe.

[3] Gupta P., Collins K.B., Ratner D., Watkins S., Naus G.J., Landers D.V., Patterson B.K. (2002). Memory cd4(+) t cells are the earliest detectable human immunodeficiency virus type 1 (hiv-1)-infected cells in the female genital mucosal tissue during hiv-1 transmission in an organ culture system. J. Virol..

[4] Zhang Z., Schuler T., Zupancic M., Wietgrefe S., Staskus K.A., Reimann K.A., Reinhart T.A., Rogan M., Cavert W., Miller C.J. (1999). Sexual transmission and propagation of siv and hiv in resting and activated cd4+ t cells. Science.

[5] Hladik F., Sakchalathorn P., Ballweber L., Lentz G., Fialkow M., Eschenbach D., McElrath M.J. (2007). Initial events in establishing vaginal entry and infection by human immunodeficiency virus type-1. Immunity.

[6] Hu J., Gardner M.B., Miller C.J. (2000). Simian immunodeficiency virus rapidly penetrates the cervicovaginal mucosa after intravaginal inoculation and infects intraepithelial dendritic cells. J. Virol..

[7] Cohen M.S., Shaw G.M., McMichael A.J., Haynes B.F. (2011). Acute hiv-1 infection. New Engl. J. Med..

[8] Reinhart T.A., Rogan M.J., Huddleston D., Rausch D.M., Eiden L.E., Haase A.T. (1997). Simian immunodeficiency virus burden in tissues and cellular compartments during clinical latency and aids. J. Infect. Dis..

[9] Schacker T., Little S., Connick E., Gebhard K., Zhang Z.Q., Krieger J., Pryor J., Havlir D., Wong J.K., Schooley R.T. (2001). Productive infection of t cells in lymphoid tissues during primary and early human immunodeficiency virus infection. J. Infect. Dis..

[10] Koenig S., Gendelman H.E., Orenstein J.M., Dal Canto M.C., Pezeshkpour G.H., Yungbluth M., Janotta F., Aksamit A., Martin M.A., Fauci A.S. (1986). Detection of aids virus in macrophages in brain tissue from aids patients with encephalopathy. Science.

[11] Desrosiers R.C., Hansen-Moosa A., Mori K., Bouvier D.P., King N.W., Daniel M.D., Ringler D.J. (1991). Macrophage-tropic variants of siv are associated with specific aids-related lesions but are not essential for the development of aids. Am. J. Pathol..

[12] Berger G., Durand S., Fargier G., Nguyen X.N., Cordeil S., Bouaziz S., Muriaux D., Darlix J.L., Cimarelli A. (2011). Apobec3a is a specific inhibitor of the early phases of hiv-1 infection in myeloid cells. PLoS Pathog..

[13] Laguette N., Rahm N., Sobhian B., Chable-Bessia C., Munch J., Snoeck J., Sauter D., Switzer W.M., Heneine W., Kirchhoff F. (2012). Evolutionary and functional analyses of the interaction between the myeloid restriction factor samhd1 and the lentiviral vpx protein. Cell Host Microbe.

[14] Geijtenbeek T.B., Kwon D.S., Torensma R., van Vliet S.J., van Duijnhoven G.C., Middel J., Cornelissen I.L., Nottet H.S., KewalRamani V.N., Littman D.R., Figdor C.G., van Kooyk Y. (2000). Dc-sign, a dendritic cell-specific hiv-1-binding protein that enhances trans-infection of t cells. Cell.

[15] Saifuddin M., Hart M.L., Gewurz H., Zhang Y., Spear G.T. (2000). Interaction of mannose-binding lectin with primary isolates of human immunodeficiency virus type 1. J. Gen. Virol..

[16] Bobardt M.D., Saphire A.C., Hung H.C., Yu X., Van der Schueren B., Zhang Z., David G., Gallay P.A. (2003). Syndecan captures, protects, and transmits hiv to t lymphocytes. Immunity.

[17] Gummuluru S., Rogel M., Stamatatos L., Emerman M. (2003). Binding of human immunodeficiency virus type 1 to immature dendritic cells can occur independently of dc-sign and mannose binding c-type lectin receptors via a cholesterol-dependent pathway. J. Virol..

[18] Izquierdo-Useros N., Lorizate M., Contreras F.X., Rodriguez-Plata M.T., Glass B., Erkizia I., Prado J.G., Casas J., Fabrias G., Krausslich H.G. (2012). Sialyllactose in viral membrane gangliosides is a novel molecular recognition pattern for mature dendritic cell capture of hiv-1. PLoS Biol..

[19] Puryear W.B., Yu X., Ramirez N.P., Reinhard B.M., Gummuluru S. (2012). Hiv-1 incorporation of host-cell-derived glycosphingolipid gm3 allows for capture by mature dendritic cells. Proc. Natl. Acad. Sci. U. S. A..

[20] Magerus-Chatinet A., Yu H., Garcia S., Ducloux E., Terris B., Bomsel M. (2007). Galactosyl ceramide expressed on dendritic cells can mediate hiv-1 transfer from monocyte derived dendritic cells to autologous t cells. Virology.

[21] de Silva S., Planelles V., Wu L. (2012). Differential effects of vpr on single-cycle and spreading hiv-1 infections in cd4+ t-cells and dendritic cells. PLoS One.

[22] Wu L., KewalRamani V.N. (2006). Dendritic-cell interactions with hiv: Infection and viral dissemination. Nat. Rev. Immunol..

[23] Cameron P.U., Freudenthal P.S., Barker J.M., Gezelter S., Inaba K., Steinman R.M. (1992). Dendritic cells exposed to human immunodeficiency virus type-1 transmit a vigorous cytopathic infection to cd4+ t cells. Science.

[24] Altfeld M., Fadda L., Frleta D., Bhardwaj N. (2011). Dcs and nk cells: Critical effectors in the immune response to hiv-1. Nat. Rev. Immunol..

[25] Coleman C.M., Wu L. (2009). Hiv interactions with monocytes and dendritic cells: Viral latency and reservoirs. Retrovirology.

[26] Sharova N., Swingler C., Sharkey M., Stevenson M. (2005). Macrophages archive hiv-1 virions for dissemination in trans. EMBO J..

[27] Lim E.S., Fregoso O.I., McCoy C.O., Matsen F.A., Malik H.S., Emerman M. (2012). The ability of primate lentiviruses to degrade the monocyte restriction factor samhd1 preceded the birth of the viral accessory protein vpx. Cell Host Microbe.

[28] Tristem M., Marshall C., Karpas A., Hill F. (1992). Evolution of the primate lentiviruses: Evidence from vpx and vpr. EMBO J..

[29] Ayinde D., Maudet C., Transy C., Margottin-Goguet F. (2010). Limelight on two hiv/siv accessory proteins in macrophage infection: Is vpx overshadowing vpr?. Retrovirology.

[30] Le Rouzic E., Benichou S. (2005). The vpr protein from hiv-1: Distinct roles along the viral life cycle. Retrovirology.

[31] Malim M.H., Emerman M. (2008). Hiv-1 accessory proteins—Ensuring viral survival in a hostile environment. Cell Host Microbe.

[32] Peeters M., Courgnaud V. (2002). Overview of primate lentiviruses and their evolution in non-human primates in Africa. HIV Sequence Compendium; HIV sequence compendium.

[33] Belshan M., Kimata J.T., Brown C., Cheng X., McCulley A., Larsen A., Thippeshappa R., Hodara V., Giavedoni L., Hirsch V. (2012). Vpx is critical for sivmne infection of pigtail macaques. Retrovirology.

[34] Hirsch V.M., Sharkey M.E., Brown C.R., Brichacek B., Goldstein S., Wakefield J., Byrum R., Elkins W.R., Hahn B.H., Lifson J.D. (1998). Vpx is required for dissemination and pathogenesis of siv(sm) pbj: Evidence of macrophage-dependent viral amplification. Nat. Med..

[35] Gibbs J.S., Lackner A.A., Lang S.M., Simon M.A., Sehgal P.K., Daniel M.D., Desrosiers R.C. (1995). Progression to aids in the absence of a gene for vpr or vpx. J. Virol..

[36] Mandal D., Prasad V.R. (2009). Analysis of 2-ltr circle junctions of viral DNA in infected cells. Meth. Mol. Biol..

[37] Sharova N., Wu Y., Zhu X., Stranska R., Kaushik R., Sharkey M., Stevenson M. (2008). Primate lentiviral vpx commandeers ddb1 to counteract a macrophage restriction. PLoS Pathog..

[38] Hrecka K., Hao C., Gierszewska M., Swanson S.K., Kesik-Brodacka M., Srivastava S., Florens L., Washburn M.P., Skowronski J. (2011). Vpx relieves inhibition of hiv-1 infection of macrophages mediated by the samhd1 protein. Nature.

[39] Laguette N., Sobhian B., Casartelli N., Ringeard M., Chable-Bessia C., Segeral E., Yatim A., Emiliani S., Schwartz O., Benkirane M. (2011). Samhd1 is the dendritic- and myeloid-cell-specific hiv-1 restriction factor counteracted by vpx. Nature.

[40] Lim E.S., Emerman M. (2011). Hiv: Going for the watchman. Nature.

[41] Planelles V. (2012). Samhd1 joins the red queen’s court. Cell Host Microbe.

[42] Baldauf H.M., Pan X., Erikson E., Schmidt S., Daddacha W., Burggraf M., Schenkova K., Ambiel I., Wabnitz G., Gramberg T. (2012). Samhd1 restricts hiv-1 infection in resting cd4(+) t cells. Nat. Med..

[43] Descours B., Cribier A., Chable-Bessia C., Ayinde D., Rice G., Crow Y., Yatim A., Schwartz O., Laguette N., Benkirane M. (2012). Samhd1 restricts hiv-1 reverse transcription in quiescent cd4+ t-cells. Retrovirology.

[44] Aravind L., Koonin E.V. (1998). The hd domain defines a new superfamily of metal-dependent phosphohydrolases. Trends Biochem. Sci..

[45] Goldstone D.C., Ennis-Adeniran V., Hedden J.J., Groom H.C., Rice G.I., Christodoulou E., Walker P.A., Kelly G., Haire L.F., Yap M.W. (2011). Hiv-1 restriction factor samhd1 is a deoxynucleoside triphosphate triphosphohydrolase. Nature.

[46] Lahouassa H., Daddacha W., Hofmann H., Ayinde D., Logue E.C., Dragin L., Bloch N., Maudet C., Bertrand M., Gramberg T. (2012). Samhd1 restricts the replication of human immunodeficiency virus type 1 by depleting the intracellular pool of deoxynucleoside triphosphates. Nat. Immunol..

[47] Jermy A. (2012). Viral infection: Samhd1 cuts the power to hiv-1. Nat. Rev. Microbiol..

[48] Schaller T., Goujon C., Malim M.H. (2012). Aids/hiv. Hiv interplay with samhd1. Science.

[49] Manel N., Hogstad B., Wang Y., Levy D.E., Unutmaz D., Littman D.R. (2010). A cryptic sensor for hiv-1 activates antiviral innate immunity in dendritic cells. Nature.

[50] Xu W., Santini P.A., Sullivan J.S., He B., Shan M., Ball S.C., Dyer W.B., Ketas T.J., Chadburn A., Cohen-Gould L. (2009). Hiv-1 evades virus-specific igg2 and iga responses by targeting systemic and intestinal b cells via long-range intercellular conduits. Nat. Immunol..

[51] Ogawa K., Shibata R., Kiyomasu T., Higuchi I., Kishida Y., Ishimoto A., Adachi A. (1989). Mutational analysis of the human immunodeficiency virus vpr open reading frame. J. Virol..

[52] Balliet J.W., Kolson D.L., Eiger G., Kim F.M., McGann K.A., Srinivasan A., Collman R. (1994). Distinct effects in primary macrophages and lymphocytes of the human immunodeficiency virus type 1 accessory genes vpr, vpu, and nef: Mutational analysis of a primary hiv-1 isolate. Virology.

[53] Rey F., BouHamdan M., Navarro J.M., Agostini I., Willetts K., Bouyac M., Tamalet C., Spire B., Vigne R., Sire J. (1998). A role for human immunodeficiency virus type 1 vpr during infection of peripheral blood mononuclear cells. J. Gen. Virol..

[54] Heinzinger N.K., Bukinsky M.I., Haggerty S.A., Ragland A.M., Kewalramani V., Lee M.A., Gendelman H.E., Ratner L., Stevenson M., Emerman M. (1994). The vpr protein of human immunodeficiency virus type 1 influences nuclear localization of viral nucleic acids in nondividing host cells. Proc. Natl. Acad. Sci. U. S. A..

[55] Lang S.M., Weeger M., Stahl-Hennig C., Coulibaly C., Hunsmann G., Muller J., Muller-Hermelink H., Fuchs D., Wachter H., Daniel M.M. (1993). Importance of vpr for infection of rhesus monkeys with simian immunodeficiency virus. J. Virol..

[56] Hoch J., Lang S.M., Weeger M., Stahl-Hennig C., Coulibaly C., Dittmer U., Hunsmann G., Fuchs D., Muller J., Sopper S. (1995). Vpr deletion mutant of simian immunodeficiency virus induces aids in rhesus monkeys. J. Virol..

[57] Somasundaran M., Sharkey M., Brichacek B., Luzuriaga K., Emerman M., Sullivan J.L., Stevenson M. (2002). Evidence for a cytopathogenicity determinant in hiv-1 vpr. Proc. Natl. Acad. Sci. U. S. A..

[58] Schrofelbauer B., Hakata Y., Landau N.R. (2007). Hiv-1 vpr function is mediated by interaction with the damage-specific DNA-binding protein ddb1. Proc. Natl. Acad. Sci. U. S. A..

[59] Kennedy E.M., Daddacha W., Slater R., Gavegnano C., Fromentin E., Schinazi R.F., Kim B. (2011). Abundant non-canonical dutp found in primary human macrophages drives its frequent incorporation by hiv-1 reverse transcriptase. J. Biol. Chem..

[60] Sire J., Querat G., Esnault C., Priet S. (2008). Uracil within DNA: An actor of antiviral immunity. Retrovirology.

[61] Norman J.M., Mashiba M., McNamara L.A., Onafuwa-Nuga A., Chiari-Fort E., Shen W., Collins K.L. (2011). The antiviral factor apobec3g enhances the recognition of hiv-infected primary t cells by natural killer cells. Nat. Immunol..

[62] Croxford J.L., Gasser S. (2011). Damage control: How hiv survives the editor apobec3g. Nat. Immunol..

[63] Harris R.S., Bishop K.N., Sheehy A.M., Craig H.M., Petersen-Mahrt S.K., Watt I.N., Neuberger M.S., Malim M.H. (2003). DNA deamination mediates innate immunity to retroviral infection. Cell.

[64] Chen R., Le Rouzic E., Kearney J.A., Mansky L.M., Benichou S. (2004). Vpr-mediated incorporation of ung2 into hiv-1 particles is required to modulate the virus mutation rate and for replication in macrophages. J. Biol. Chem..

[65] Zimmerman E.S., Sherman M.P., Blackett J.L., Neidleman J.A., Kreis C., Mundt P., Williams S.A., Warmerdam M., Kahn J., Hecht F.M., Grant R.M., de Noronha C.M., Weyrich A.S., Greene W.C., Planelles V. (2006). Human immunodeficiency virus type 1 vpr induces DNA replication stress *in vitro* and *in vivo*. J. Virol..

[66] Lavin M.F., Kozlov S. (2007). Atm activation and DNA damage response. Cell Cycle.

[67] Cimprich K.A., Cortez D. (2008). Atr: An essential regulator of genome integrity. Nat. Rev. Mol. Cell Biol..

[68] Priet S., Gros N., Navarro J.M., Boretto J., Canard B., Querat G., Sire J. (2005). Hiv-1-associated uracil DNA glycosylase activity controls dutp misincorporation in viral DNA and is essential to the hiv-1 life cycle. Mol. Cell.

[69] Gu Y., Sundquist W.I. (2003). Good to cu. Nature.

[70] Yang B., Chen K., Zhang C., Huang S., Zhang H. (2007). Virion-associated uracil DNA glycosylase-2 and apurinic/apyrimidinic endonuclease are involved in the degradation of apobec3g-edited nascent hiv-1 DNA. J. Biol. Chem..

[71] Yan N., O'Day E., Wheeler L.A., Engelman A., Lieberman J. (2011). Hiv DNA is heavily uracilated, which protects it from autointegration. Proc. Natl. Acad. Sci. U. S. A..

[72] Sheehy A.M., Gaddis N.C., Choi J.D., Malim M.H. (2002). Isolation of a human gene that inhibits hiv-1 infection and is suppressed by the viral vif protein. Nature.

[73] Bishop K.N., Verma M., Kim E.Y., Wolinsky S.M., Malim M.H. (2008). Apobec3g inhibits elongation of hiv-1 reverse transcripts. PLoS Pathog..

[74] Koning F.A., Newman E.N., Kim E.Y., Kunstman K.J., Wolinsky S.M., Malim M.H. (2009). Defining apobec3 expression patterns in human tissues and hematopoietic cell subsets. J. Virol..

[75] Malim M.H. (2009). Apobec proteins and intrinsic resistance to hiv-1 infection. Phil. Trans. Roy. Soc. Lond. B Biol. Sci..

[76] Peng G., Greenwell-Wild T., Nares S., Jin W., Lei K.J., Rangel Z.G., Munson P.J., Wahl S.M. (2007). Myeloid differentiation and susceptibility to hiv-1 are linked to apobec3 expression. Blood.

[77] Koning F.A., Goujon C., Bauby H., Malim M.H. (2011). Target cell-mediated editing of hiv-1 cdna by apobec3 proteins in human macrophages. J. Virol..

[78] Chiu Y.L., Greene W.C. (2008). The apobec3 cytidine deaminases: An innate defensive network opposing exogenous retroviruses and endogenous retroelements. Ann. Rev. Immunol..

